# Distinct Pathogenic Mechanisms of Two Novel NHS Mutations Identified in Chinese Han Families With Nance–Horan Syndrome

**DOI:** 10.1155/humu/3739907

**Published:** 2026-05-28

**Authors:** Li Li, Jiaxi Song, Meiling Qin, Shuyu Zhou, Jingfan Liu, Guangying Zheng

**Affiliations:** ^1^ Eye Center, The First Affiliated Hospital of Zhengzhou University, Zhengzhou, Henan Province, China, zzu.edu.cn

**Keywords:** congenital cataract, extracellular matrix disruption, gene mutation, mitochondrial dysfunction, Nance–Horan syndrome

## Abstract

Nance–Horan syndrome (NHS) is a rare X‐linked genetic disorder characterized by congenital cataracts, dental anomalies, and neurodevelopmental impairments, caused by mutations in the *NHS* gene. In this study, two novel *NHS* mutations, c.3847C>T and c.2519_2520del, were identified in two unrelated Chinese Han families, and their pathogenic molecular mechanisms were elucidated. Functional analyses revealed that the c.3847C>T mutation exerts a dual pathogenic effect: It disrupts extracellular matrix homeostasis by downregulating *COL4A1* and upregulating *MMP9*, and it triggers oxidative stress, evidenced by elevated ROS levels, altered BAX/BCL‐2 ratio, and reduced *GPX4* expression, ultimately leading to apoptosis and impaired cell migration. In contrast, the c.2519_2520del mutation primarily impairs mitochondrial function, as indicated by decreased membrane potential, reduced ATP production, and downregulation of *ALDH3A1* and *SOD2*. This mitochondrial dysfunction is further exacerbated by suppressed *PGC-1α* expression, contributing to metabolic disturbances, and by *p21*‐mediated cell cycle arrest. These findings, for the first time, demonstrate that distinct types of *NHS* mutations cause disease via divergent mechanisms, extracellular matrix disruption versus mitochondrial dysfunction, providing molecular insights into the clinical phenotypic heterogeneity of NHS and laying a theoretical foundation for the development of mutation‐specific precision therapies.

## 1. Introduction

Nance–Horan syndrome (NHS), also referred to as cataract‐dental syndrome, is a rare X‐linked developmental disorder first described in 1974 [1]. Clinically, NHS is characterized by a distinctive triad comprising neurological abnormalities, ocular defects, and dental and craniofacial anomalies. The most penetrant ocular feature is bilateral dense congenital cataracts, which frequently result in severe visual impairment and often require early surgical intervention [2]. Neurological involvement may manifest as intellectual disability, developmental delay, or autism spectrum‐related features, while dental anomalies such as screwdriver‐shaped incisors and mulberry molars remain hallmark signs of the condition [2]. In addition, several characteristic craniofacial and skeletal features have been reported, including a long, narrow face, anteverted pinnae, a broad nasal bridge, and shortened metacarpals [3].

The genetic basis of NHS lies in pathogenic variants of the NHS gene located on chromosome Xp22.13, which encodes a large protein implicated in the regulation of epithelial polarity, actin cytoskeleton organization, and cell–cell adhesion [4]. Disruption of these cellular processes can have widespread developmental consequences, consistent with the involvement of multiple tissues such as the lens, enamel epithelium, and neuronal systems. Loss‐of‐function mutations, including nonsense, frameshift, and splice‐site variants, have been shown to cause protein truncation and dysfunction, thereby producing the diverse phenotypic spectrum of NHS [5, 6]. Clinical severity differs substantially between sexes: Hemizygous males typically exhibit severe ocular malformations, including microcornea and nystagmus, as well as systemic anomalies, while female carriers often display only mild features such as posterior Y‐suture cataracts. This sex‐based variability is thought to be influenced by patterns of X‐chromosome inactivation [7, 8].

To date, more than 60 pathogenic *NHS* variants have been documented, with most clustering within Exon 6, a region likely critical for protein function. This region likely encodes a functionally critical domain of the protein, and its disruption appears to play a key role in disease pathogenesis [9, 10]. Interestingly, the distribution of NHS mutations appears to vary between populations [11]. In Chinese cohorts, relatively few studies have been reported, yet population‐specific variants have already been described. For example, in 2014, a four‐generation Chinese pedigree carrying a nonsense mutation in Exon 1 (c.322G>T, p.E108X) was reported, which led to premature protein truncation and impaired cell migration [12]. More recently, in 2025, a frameshift mutation in Exon 6 (c.1735delA, p.R579Gfs ^∗^91) was identified, further expanding the clinical spectrum of NHS in the Chinese population. Notably, in addition to typical ocular and dental anomalies, affected patients also presented with rare comorbidities such as congenital heart disease [13].

Despite the increasing number of reported NHS variants, the mechanistic links between mutation type and downstream pathogenic pathways remain poorly understood. Current studies largely emphasize variant identification and genotype–phenotype correlations, but relatively few have explored how different classes of mutations perturb NHS protein function at the molecular and cellular levels. This gap in knowledge limits our understanding of why patients with different NHS mutations can present with heterogeneous clinical outcomes.

In our previous clinical and genetic screening of two unrelated Chinese Han families diagnosed with NHS, we identified two novel Exon 6 variants: (1) c.3847C>T (p.Gln1283 ^∗^), a nonsense mutation predicted to cause premature translational termination, and (2) c.2519_2520del (p.Ser840fs), a frameshift mutation resulting in protein truncation [14]. Both mutations segregated with disease in an X‐linked inheritance pattern and were detected in hemizygous male probands. In the present study, we established CRISPR‐Cas9‐engineered cell models to systematically evaluate the molecular and cellular consequences of these two mutations. Using transcriptomic profiling and functional assays, we demonstrate that the c.3847C>T mutation primarily disrupts extracellular matrix homeostasis and induces oxidative stress, whereas the c.2519_2520del mutation mainly impairs mitochondrial metabolism and energy balance. These findings provide new mechanistic insights into how different classes of NHS mutations contribute to disease via divergent molecular pathways, thereby laying the groundwork for the development of mutation‐specific therapeutic strategies.

## 2. Materials and Methods

### 2.1. Cell and Bacterial Strains

The human lens epithelial cell line B3 was obtained from the BeNa Culture Collection (supplied by Guangzhou Zuoke Biotechnology Development Co. Ltd., China) on March 4, 2024. This cell line was selected due to its direct disease relevance to NHS‐associated congenital cataracts, as it originates from the human lens epithelium, the primary tissue affected by NHS. B3 cells inherently express the NHS gene, providing a biologically appropriate model to investigate the functional impact of NHS mutations on lens development and disease mechanisms. Authentication was performed by short tandem repeat (STR) profiling, and the cells tested negative for mycoplasma contamination.

Cells were cultured in Eagle′s Minimum Essential Medium (EMEM) supplemented with 10% fetal bovine serum (FBS), 100 U/mL penicillin, and 100 *μ*g/mL streptomycin at 37°C in a humidified incubator containing 5% CO_2_. Chemically competent *Escherichia coli* DH5*α* cells were obtained from Vazyme Biotech Co. Ltd. (Nanjing, China). The LentiCRISPRv2‐GFP vector was purchased from MiaoLingBio (Beijing, China).

### 2.2. Generation of NHS‐Mutant HLE‐B3 Cell Lines

Based on the reference sequence of the *NHS* gene (NM_198270.4) from the NCBI database, specific single‐guide RNAs (sgRNAs) targeting the c.3847C>T and c.2519_2520del mutation sites were designed using the CRISPOR online tool (https://crispor.gi.ucsc.edu/) (sgRNA sequences listed in Table S1). sgRNA oligonucleotides were annealed and ligated into BsaI‐digested LentiCRISPRv2‐GFP vectors. The recombinant plasmids were transformed into DH5*α* competent cells, plated on LB agar containing 100 *μ*g/mL ampicillin, and incubated at 37°C for 16 h. Single colonies were screened by PCR and validated by Sanger sequencing. Verified recombinant plasmids were extracted and transfected into HLE‐B3 cells using Lipofectamine 3000 (Thermo Fisher Scientific, United States). After 48 h, GFP‐positive cells were sorted by flow cytometry and expanded for subsequent experiments.

### 2.3. Gene Mutation Validation

Following CRISPR‐Cas9‐mediated knockout, genomic DNA was extracted from both mutant and wild‐type control cells using a commercial DNA extraction kit. The target region containing the mutation site was amplified by PCR (primers listed in Table S2), and the PCR products were verified by 2% agarose gel electrophoresis to confirm the expected amplicon size. The purified PCR products were subsequently subjected to Sanger sequencing, and the resulting chromatograms were analyzed using SnapGene software to confirm successful mutation incorporation.

### 2.4. RNA‐Seq Analysis of NHS‐Mutant Cell Lines

RNA extraction, library preparation, and sequencing were performed by Frasergen (Wuhan, China) using the MGI HiSeq platform. Gene expression levels were quantified using the RSEM software suite. Differentially expressed genes (DEGs) were identified using the DESeq2 algorithm. To analyze enriched biological pathways, Gene Ontology (GO) and Kyoto Encyclopedia of Genes and Genomes (KEGG) analyses were conducted using Goseq and KOBAS 3.0, respectively. Visualization of Venn diagrams and heatmaps was performed using HiPlot (https://hiplot.cn/).

### 2.5. Cell Proliferation Assay

Wild‐type and NHS‐mutant HLE‐B3 cells in the logarithmic growth phase were seeded into 96‐well plates at a density of 1 × 10^4^ cells/well and cultured for 24 h. Subsequently, 10 *μ*L of CCK‐8 solution (Beyotime, China) was added to each well and incubated for 1 h. The absorbance at 450 nm was measured using a microplate reader, and the relative proliferation rate was calculated.

### 2.6. Measurement of Reactive Oxygen Species (ROS)

Cells were washed three times with PBS, followed by incubation with 10 *μ*M DCFH‐DA probe (Beyotime, China) at 37°C in the dark for 30 min. After washing three times with serum‐free medium, cells were either imaged using a laser scanning confocal microscope (LSM 880, Zeiss, Germany) or resuspended in PBS for flow cytometric analysis (Beckman Coulter, United States).

### 2.7. Cell Cycle Analysis

Cells were trypsinized, washed once with prechilled PBS, and fixed in 2 mL of 70% ethanol at −20°C for 30 min. After washing with PBS, cells were incubated with 20 *μ*g/mL RNase A at 37°C for 30 min, resuspended in 500 *μ*L PBS containing 25 *μ*L propidium iodide (PI; Yeasen, China), and stained on ice in the dark for 30 min. Samples were filtered through a 300‐mesh nylon sieve prior to flow cytometric analysis (Beckman Coulter, United States).

### 2.8. Apoptosis Assay

Both adherent and floating cells were collected, washed with PBS, and digested with trypsin without EDTA. The digestion was stopped by adding complete medium, followed by centrifugation and two washes with prechilled PBS. Apoptotic cells were detected using the Annexin V‐FITC/PI Apoptosis Detection Kit (Biosharp, China) according to the manufacturer′s instructions and analyzed by flow cytometry. Data were processed using FlowJo software. All experiments were performed in triplicate.

### 2.9. Wound Healing Assay

Wild‐type and NHS‐mutant HLE‐B3 cells in the logarithmic growth phase were seeded into six‐well plates (2 × 10^5^ cells/well) with reference lines premarked on the plate bottom. After 24 h, when cells reached full confluence, the culture medium was replaced with low‐serum medium (containing 1% FBS) to inhibit cell proliferation. Two parallel scratches perpendicular to the reference lines were made using a sterile 200 *μ*L pipette tip. Detached cells were removed by PBS washing, and fresh 1% FBS medium was added. Images were captured at 0, 24, and 48 h using an inverted microscope. Wound closure was quantified using ImageJ software.

### 2.10. Mitochondrial Membrane Potential Assay

Cells were stained using the JC‐1 assay kit (Beyotime, China) according to the manufacturer′s protocol. Fluorescence was observed immediately under a fluorescence microscope, with excitation at 488 nm and emission at 530 nm (monomer) and 590 nm (aggregate).

### 2.11. ATP Quantification

After 24 h of culture, the medium was removed, and cells were washed once with PBS. Intracellular ATP levels were measured using the Enhanced ATP Assay Kit (Beyotime, China) according to the manufacturer′s instructions. Luminescence (RLU) was detected using a luminometer, and ATP concentrations were calculated from a standard curve and normalized to total protein content (nanomoles per milligram of protein).

### 2.12. RNA Extraction and Quantitative PCR (qPCR)

Total RNA was extracted using TRIzol reagent (Invitrogen, United States) and reverse transcribed into cDNA using the HiScript II Q RT SuperMix for qPCR kit (Vazyme, China). *β*‐Actin served as the internal reference. qPCR was performed with AceQ qPCR SYBR Green Master Mix (Vazyme, China) on a real‐time PCR system. Each sample included three biological replicates, each with three technical replicates. Relative gene expression was calculated using the 2^−*Δ* *Δ*Ct^ method. Primers are listed in Table S3.

### 2.13. Measurement of Malondialdehyde (MDA), Superoxide Dismutase (SOD), and Catalase (CAT) Activities

Wild‐type and NHS‐mutant HLE‐B3 cells were seeded into six‐well plates (2 × 10^5^ cells/well) and cultured for 24 h. Cells were washed with prechilled PBS, lysed, and centrifuged at 12,000 × g for 10 min at 4°C. MDA levels were measured using the thiobarbituric acid method (Beyotime, China) at 532 nm. SOD activity was determined using the WST‐8 method (Beyotime, China) at 450 nm. CAT activity was measured by monitoring the reaction rate at 240 nm.

### 2.14. Statistical Analysis

All experiments were performed with at least three biological replicates. Data are presented as mean ± standard deviation (SD). Statistical analyses were conducted using GraphPad Prism 8. Differences between the two groups were assessed by Student′s *t*‐test. Comparisons among multiple groups were performed using one‐way ANOVA followed by Tukey′s post hoc test. A *p* value < 0.05 ( ^∗^), or < 0.01 ( ^∗∗^),or < 0.001 (***), or < 0.0001 (****) was considered statistically significant.

## 3. Results

### 3.1. Identification and Transcriptomic Profiling of NHS Exon 6 Mutations

The identity of the B3 human lens epithelial cell line was verified by STR genotyping, which showed a matching rate ≥ 80% with the reference database, confirming its authenticity. In addition, mycoplasma testing was negative, ensuring that all subsequent experiments were conducted with contamination‐free cells. The reliable authentication and aseptic status of this cell line strengthen the reproducibility of the experimental results. This study successfully established two NHS gene Exon 6 mutant cell models using CRISPR‐Cas9 technology for subsequent research (Figure 1A; sgRNA and ssODN donor sequences are listed in Table S1). The site mutation (c.3847C>T) introduced a premature termination codon, while the Del mutation (c.2519_2520del) caused frameshift translation. Sanger sequencing confirmed successful mutation incorporation in both models (Figure S1). To determine the molecular impact of these PTCs, we performed RT‐qPCR analysis. The results revealed a significant reduction in NHS mRNA expression in both mutant lines compared to wild‐type cells, with a more pronounced decrease in the Del mutation (Figure S2), suggesting the occurrence of nonsense‐mediated mRNA decay (NMD). Transcriptome analysis revealed that compared to wild‐type cells, the site mutation resulted in 1070 DEGs (Figure 1B). Volcano plot analysis demonstrated that the site mutation induced 229 upregulated and 841 downregulated genes (Figure S3). Functional enrichment analysis showed that the downregulated genes were significantly enriched in cell–matrix adhesion and tight junction‐related pathways (Figure 1C), whereas the upregulated genes were primarily associated with oxidative stress‐related processes (Figure 1D). These findings suggest that the site mutation may disrupt cell polarity connections and trigger oxidative stress and ECM imbalance [15–17].

Figure 1Identification of NHS mutations and transcriptomic analysis. (A) Schematic of NHS mutant construction strategy (c.3847C>T and c.2519_2520del). (B) Heatmap of differentially expressed genes (|log2FC| > 1, FDR < 0.05). (C, D) KEGG pathway enrichment analysis and GO term enrichment analysis of site mutations. (E, F) KEGG pathway enrichment analysis and GO term enrichment analysis of Del mutations. c.3847C>T (site mutation) and c.2519_2520del (Del mutation).

**Figure:**
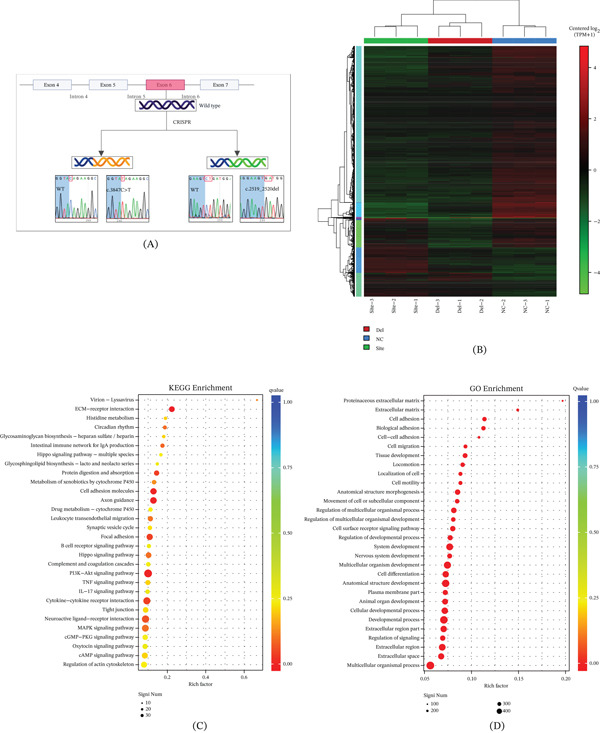
(A)

**Figure:**
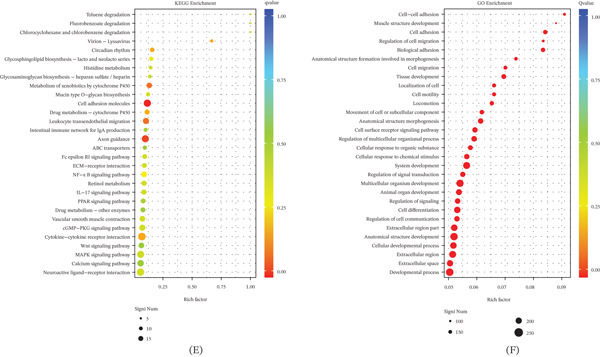
(B)

In contrast, the Del mutation led to 780 DEGs (Figure 1)B. Volcano plot analysis identified 81 upregulated and 699 downregulated genes (Figure S3). The downregulated gene set showed significant enrichment in mitochondrial metabolism–related pathways, characterized by a widespread reduction in mitochondrial function‐related genes (Figure 1E,F). These findings indicate that the Del mutation primarily affects cellular function by interfering with mitochondrial metabolic homeostasis [18, 19]. The distinct transcriptional alterations between these two mutations provide important molecular mechanistic explanations for subsequent phenotypic studies.

### 3.2. Cellular Phenotypic Effects of *NHS* Mutations

**Figure 2:**
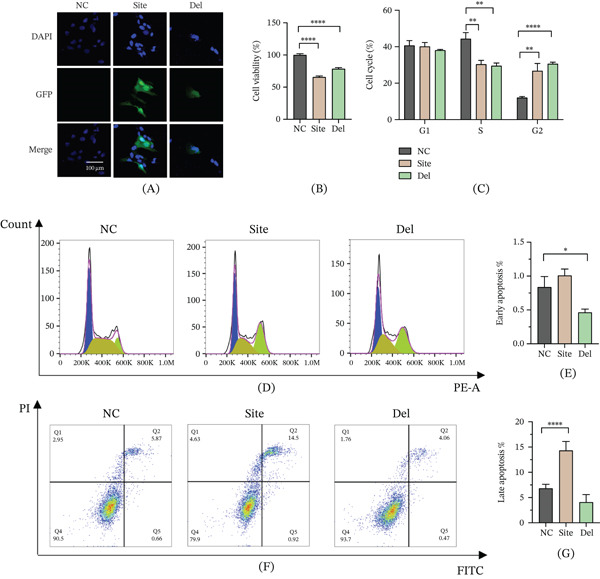
Effects of NHS mutations on cell survival and cell cycle progression. (A) Transfection efficiency of GFP‐tagged mutants analyzed by confocal microscopy (scale bar: 100 *μ*m). (B) Impact of mutation on cell proliferation assessed by CCK‐8 assay (mean ± SD, *n* = 3). (C, D) Cell cycle distribution in mutant versus control groups quantified by flow cytometry (percentages of G1, S, and G2 phases). (E–G) Mutation‐induced apoptosis measured by Annexin V‐FITC/PI double staining. c.3847C>T (site mutation) and c.2519_2520del (Del mutation).

Using confocal microscopy and flow cytometry, we assessed the impact of *NHS* mutations on basic cellular phenotypes. Transfection efficiency, monitored by GFP fluorescence, was higher in cells with the c.3847C>T mutation compared to those with the c.2519_2520del mutation (Figure 2A). GFP‐positive cells were sorted by flow cytometry to ensure pure populations for downstream assays.

CCK‐8 proliferation assays demonstrated significantly reduced growth rates in both mutant cell lines compared to wild‐type controls (Figure 2B). Cell cycle analysis revealed a decrease in the S‐phase population and an accumulation of cells in the G2 phase (Figure 2C,D), indicating cell cycle arrest. Annexin V/PI staining showed mutation‐specific apoptotic responses; the c.3847C>T mutant exhibited markedly increased apoptosis, consistent with the transcriptomic upregulation of genes associated with oxidative stress and the proapoptotic pathway (Figure 2E–G), suggesting apoptosis is driven by oxidative damage [20, 21]. Meanwhile, the c.2519_2520del mutant displayed apoptosis rates comparable to wild‐type but notable G2‐phase arrest, correlating with the downregulation of key mitochondrial and metabolic regulators (Figure 2C,D), implicating mitochondrial dysfunction in cell cycle disruption [22]. These findings support the transcriptome‐predicted divergent mechanisms, whereby the c.3847C>T mutation triggers apoptosis via oxidative stress and ECM imbalance, and the c.2519_2520del mutation disrupts cell cycle progression through impaired mitochondrial metabolism.

### 3.3. Oxidative Stress and Mitochondrial Dysfunction in Mutant Cells

**Figure 3:**
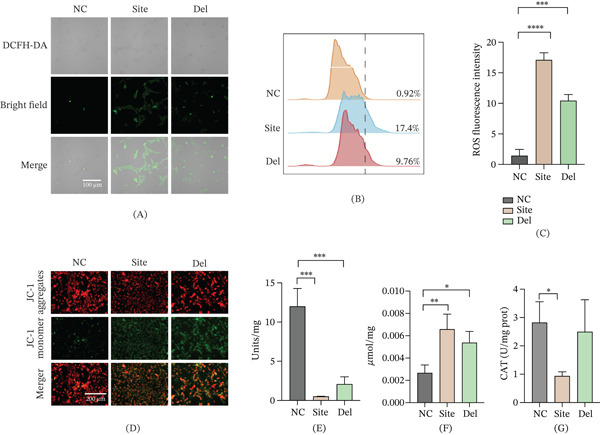
NHS mutation–induced oxidative stress and mitochondrial dysfunction. Intracellular ROS level detection in mutants: (A) confocal microscopy of DCFH‐DA fluorescence intensity (scale bar: 200 *μ*m), (B) flow cytometric quantification of ROS‐positive cells, and (C) statistical analysis of ROS levels. (D) JC‐1 staining assessing mitochondrial membrane potential (red/green fluorescence ratio decrease indicates depolarization). Oxidative stress and energy metabolism parameters: (E) lipid peroxidation product MDA content, (F) antioxidant enzyme SOD activity, and (G) intracellular ATP levels (mean ± SEM, *n* = 3;  ^∗^ *p* < 0.05,  ^∗∗^ *p* < 0.01,  ^∗∗∗^ *p* < 0.001, and  ^∗∗∗∗^ *p* < 0.001).

Assessment of intracellular ROS levels using the DCFH‐DA probe revealed significant increases in both mutant groups compared to controls (Figure 3A–C), confirming induction of oxidative stress by the *NHS* mutations. JC‐1 staining demonstrated a decreased red/green fluorescence ratio in both mutants (Figure 3D), indicative of mitochondrial membrane depolarization and dysfunction.

Biochemical assays further corroborated oxidative damage: MDA levels were significantly elevated (Figure 3E), reflecting enhanced lipid peroxidation, while SOD activity was markedly reduced (Figure 3F), indicating compromised antioxidant defenses. Additionally, intracellular ATP content was substantially decreased in mutant cells (Figure 3G), confirming mitochondrial energy metabolism impairment. Collectively, these data show that both the c.3847C>T and c.2519_2520del mutations induce oxidative stress and mitochondrial dysfunction, culminating in cellular metabolic imbalance and a predisposition to apoptosis.

### 3.4. Differential Effects of *NHS* Mutations on Cell Migration and Molecular Pathways

**Figure 4:**
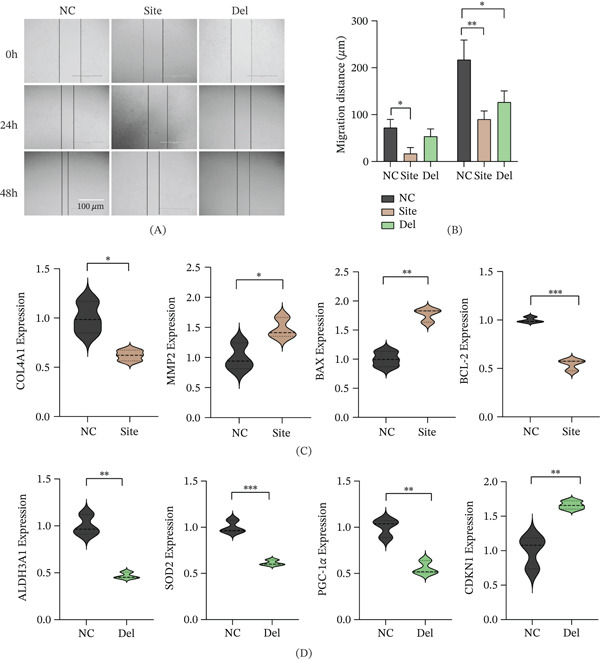
NHS mutation–induced migration defects and associated molecular alterations. Wound healing assay assessing cell migration capacity: (A) representative images at 0 and 24 h (dashed lines indicate wound edges) and (B) quantification of linear migration distance. qRT‐PCR analysis of differentially expressed genes: (C) mRNA levels of ECM‐related genes (COL4A1, MMP9, BAX, and BCL‐2) in site mutant (c.3847C>T) and (D) mRNA levels of metabolism‐related genes (ALDH3A1, SOD2, PGC‐1*α*, and CDKN1) in Del mutant (c.2519_2520del) (mean ± SEM, *n* = 3;  ^∗^ *p* < 0.05,  ^∗∗^ *p* < 0.01, and  ^∗∗∗^ *p* < 0.001).

Wound healing assays revealed that cell migration was significantly impaired in both mutant lines compared to wild‐type cells (Figure 4A,B). To investigate the underlying molecular mechanisms, we selected key representative markers from the most significantly enriched pathways in our transcriptomic data for validation. qPCR analysis indicated that within the ECM‐related gene cluster, the c.3847C>T mutation caused significant downregulation of the essential basement membrane component *COL4A1* and upregulation of matrix metalloproteinase *MMP2* (Figure 4C), indicative of disrupted ECM homeostasis [23, 24]. Furthermore, proapoptotic *BAX* expression was elevated with concurrent reduction of antiapoptotic *BCL-2*, consistent with increased apoptotic rates observed earlier.

Conversely, focusing on mitochondrial metabolism, the c.2519_2520del mutation significantly reduced expression of the core antioxidant gene *SOD2* and the master mitochondrial biogenesis regulator *PGC-1α* (Figure 4D), consistent with the observed mitochondrial dysfunction phenotype [25]. These markers were prioritized for validation due to their high fold‐change values and established roles as hub regulators in their respective pathways. The cell cycle inhibitor *CDKN1A* (*p21*) was also markedly upregulated, providing a molecular explanation for the G2 phase arrest detected.

In summary, while both mutations impair cell migration, their underlying molecular mechanisms differ: The c.3847C>T mutation primarily disrupts ECM homeostasis and activates apoptosis pathways, whereas the c.2519_2520del mutation predominantly causes mitochondrial metabolic disturbances and cell cycle arrest. These findings provide novel experimental evidence elucidating how distinct *NHS* mutations differentially compromise cellular function and contribute to disease pathogenesis.

## 4. Discussion

In this study, we established two NHS Exon 6 mutant cell models and demonstrated that distinct mutations lead to divergent molecular perturbations despite converging on oxidative stress and impaired migration. The nonsense mutation c.3847C>T primarily disrupted ECM homeostasis and triggered oxidative stress–driven apoptosis, whereas the frameshift mutation c.2519_2520del predominantly impaired mitochondrial metabolism and induced p21‐mediated cell cycle arrest. These findings not only reveal mutation‐specific pathogenic mechanisms but also provide insights into the phenotypic heterogeneity of NHS.

Previous studies have suggested that NHS protein plays an essential role in maintaining cytoskeletal organization, intercellular junctions, and lens fiber integrity [26–28]. Our transcriptomic and phenotypic analyses extend this knowledge by demonstrating that the c.3847C>T mutation downregulates adhesion‐ and tight junction‐related genes (CLDN3 and ZO‐1) while upregulating oxidative stress mediators (HMOX1 and NOX4). These alterations synergistically compromise ECM stability and cellular redox balance, leading to apoptosis. Dysregulated ECM remodeling, evidenced by decreased COL4A1 and increased MMP9 expression, may not only hinder lens transparency but also contribute to neurodevelopmental abnormalities through impaired neuronal adhesion and survival [29, 30]. This mechanism aligns with prior observations that ECM defects and oxidative stress exacerbate cataractogenesis and may underlie broader systemic manifestations of NHS [31, 32].

By contrast, the c.2519_2520del mutation exerted its pathogenicity primarily through mitochondrial dysfunction. The significant downregulation of mitochondrial respiratory chain components (ATP5F1 and NDUFA6) and antioxidant enzymes (SOD2 and ALDH3A1) led to decreased ATP levels and mitochondrial membrane depolarization. Mitochondrial defects are known contributors to cataract formation and systemic developmental abnormalities [19, 33]. Importantly, the observed upregulation of CDKN1A (p21) indicates that mitochondrial dysfunction may trigger cell cycle checkpoint activation, thereby causing G2 phase arrest and reduced proliferation. Such defects in energy metabolism and cell cycle regulation could underlie the growth retardation and neurodevelopmental delays reported in NHS patients. These findings highlight a previously underappreciated connection between NHS mutations and mitochondrial metabolic regulation, which may contribute to the neurological features of the disease.

Although both mutations ultimately induced oxidative stress, mitochondrial dysfunction, and impaired migration, their upstream mechanisms were distinct. This divergence may explain why NHS patients present with heterogeneous phenotypes ranging from isolated congenital cataracts to syndromic features, including intellectual disability and dysmorphic facies [34, 35]. The c.3847C>T mutation′s emphasis on ECM disruption and apoptosis may be more closely associated with ocular and craniofacial phenotypes, while the c.2519_2520del mutation′s link to metabolic dysfunction and cell cycle arrest may preferentially affect systemic growth and neurological outcomes. These results underscore the necessity of considering mutation‐specific mechanisms when interpreting genotype–phenotype correlations in NHS.

From a translational standpoint, our findings provide a rationale for mutation‐stratified therapeutic strategies. For patients with ECM‐related defects (such as c.3847C>T), treatments aimed at stabilizing ECM integrity or reducing oxidative stress, such as antioxidants, MMP inhibitors, or ferroptosis modulators, may mitigate disease progression [36–38]. Conversely, for mutations causing mitochondrial dysfunction (such as c.2519_2520del), therapeutic strategies enhancing mitochondrial biogenesis or metabolic support, including NAD+ precursors and PGC‐1*α* activators, may hold promise [39, 40]. This mutation‐based approach reflects the broader trend toward precision medicine in hereditary eye diseases, where targeted therapies are tailored to the specific pathogenic mechanism.

Several limitations of this study should be acknowledged. First, the use of in vitro cell models may not fully recapitulate the complex tissue‐specific context of the NHS, particularly in lens and neuronal development. Validation in animal models and patient‐derived samples will be necessary to confirm these mechanisms. Second, the precise interaction network of the NHS protein remains incompletely defined. Future studies integrating proteomic mapping and functional assays will be crucial to delineate how NHS mutations disrupt protein complexes involved in cytoskeletal organization, ECM remodeling, and mitochondrial regulation.

## 5. Conclusion

**Figure 5:**
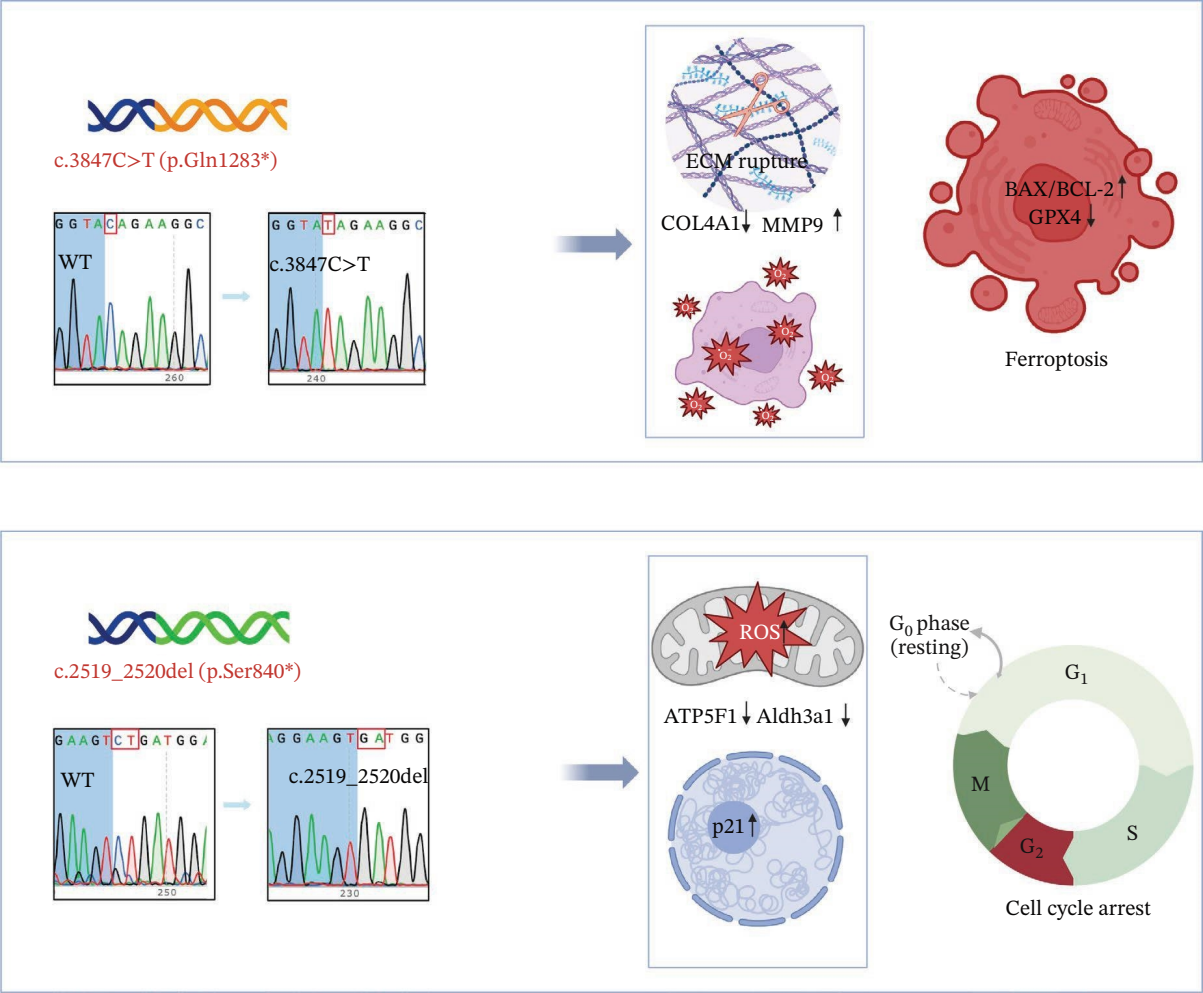
Differential pathogenic mechanisms of NHS mutations in cellular dysfunction.

In summary, this study identified two novel NHS gene mutations, c.3847C>T (p.Gln1283 ^∗^) and c.2519_2520del (p.Ser840fs), that cause NHS through distinct molecular mechanisms. The c.3847C>T mutation primarily disrupts extracellular matrix homeostasis and induces oxidative stress, leading to apoptosis and impaired cell migration. In contrast, the c.2519_2520del mutation predominantly impairs mitochondrial function and energy metabolism, resulting in cell cycle arrest and metabolic dysfunction. These divergent pathogenic pathways provide a molecular basis for the clinical heterogeneity of NHS and underscore the importance of mutation‐specific therapeutic strategies (Figure 5).

## Author Contributions

**Li Li:** conceptualization, methodology, supervision, project administration, writing – original draft, writing – review and editing. **Jiaxi Song:** visualization, validation, methodology. **Meiling Qin:** methodology, validation. **Shuyu Zhou:** visualization, formal analysis, resources. **Jingfan Liu:** methodology, resources. **Guangying Zheng:** writing – review and editing.

## Funding

This study was funded by the National Natural Science Foundation of China (82000875) and the Provincial Science and Technology Research and Development Plan Joint Fund Projects of Henan Province, China (Key Projects) (225200810022).

## Conflicts of Interest

The authors declare no conflicts of interest.

## Supporting information


**Supporting Information** Additional supporting information can be found online in the Supporting Information section. Supporting Information for this work can be found in e‐version of this paper online.

## Data Availability

Data will be made available upon request.
