# Supplementary material for: Distinct Pathogenic Mechanisms of Two Novel NHS Mutations Identified in Chinese Han Families With Nance–Horan Syndrome
Source: Hum Mutat. 2026 May 28;2026:3739907. doi: 10.1155/humu/3739907 (PMC13240491; doi:10.1155/humu/3739907)
Supplement: Supplementary file 1 — Supporting Information Additional supporting information can be found online in the Supporting Information section. Supporting Information for this work can be found in e‐version of this paper online. [file HUMU-2026-3739907-s001.docx]

**Distinct pathogenic mechanisms of two novel NHS mutations identified in Chinese Han families with Nance-Horan syndrome**

Li Li*, Jiaxi Song, Meiling Qin, Shuyu Zhou, Jingfan Liu, Guangying Zheng

Eye Center, The First Affiliated Hospital of Zhengzhou University, Zhengzhou 450052, Henan Province, China

*Corresponding author:

Dr. Li Li (E-mail: redlily625@126.com)

**
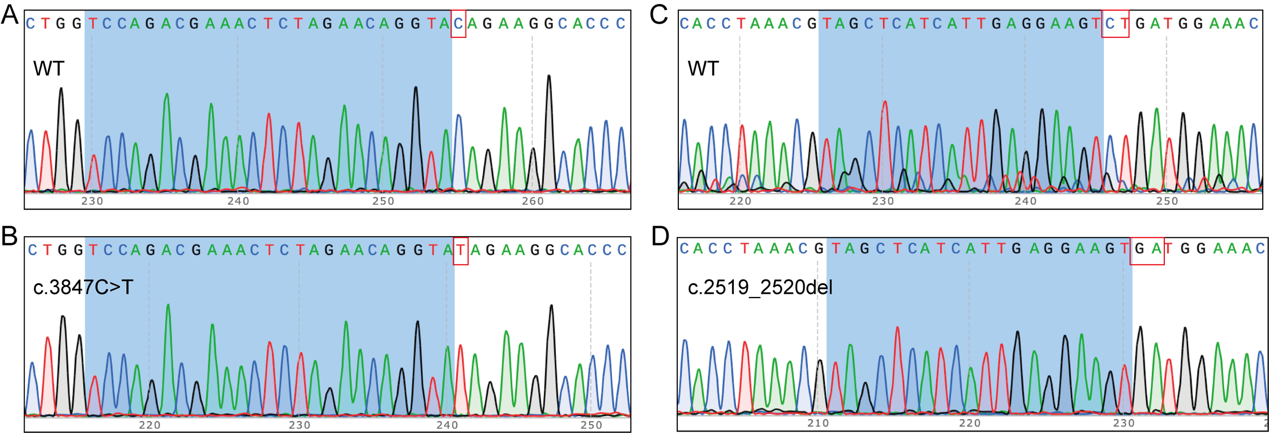
**

**Figure S1.** Sanger sequencing validation of the CRISPR-Cas9-mediated NHS mutations. (A, C) Sequencing chromatograms of the wild-type (WT) alleles corresponding to the targeted sites. (B) Sequencing chromatogram of the c.3847C>T mutant allele indicated by the red box. (D) Sequencing chromatogram of the c.2519_2520del mutant allele indicated by the red box.


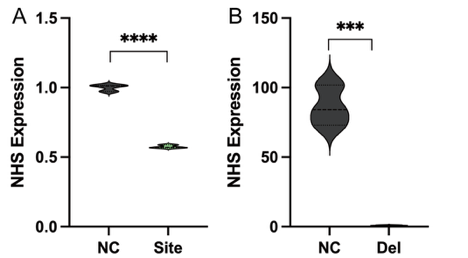


**Figure S2.** RT-qPCR validation of *NHS* mRNA expression in wild and mutant HLE-B3 cell lines.

**
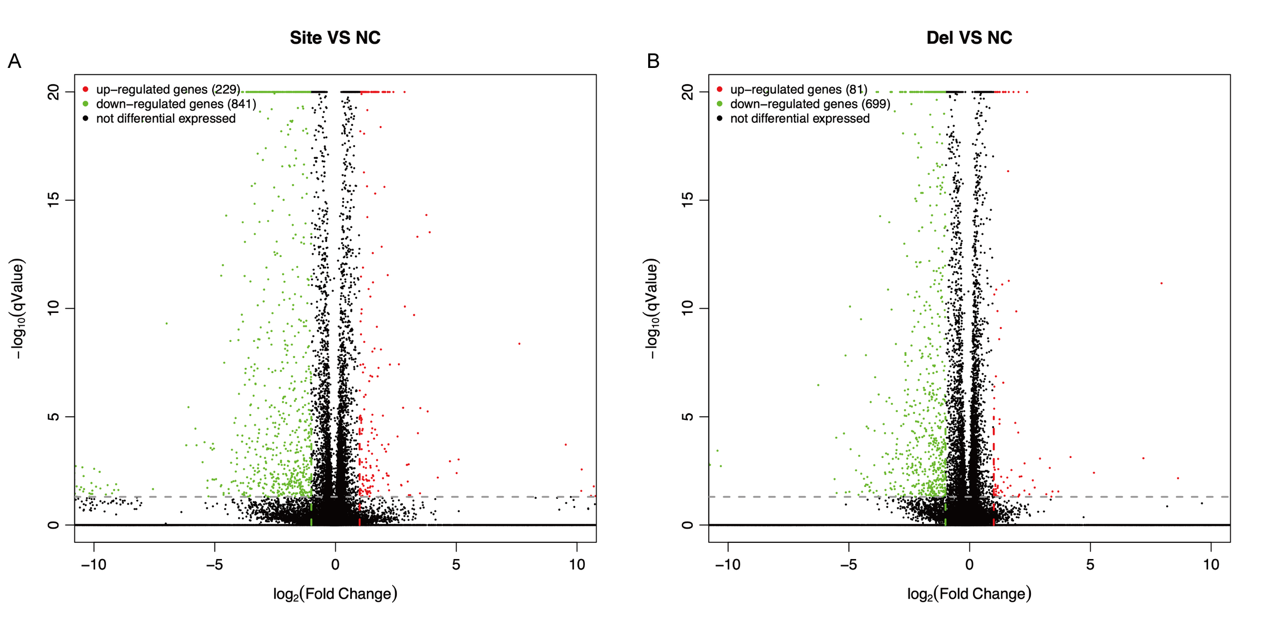
**

**Figure S3.** Differential gene expression profiles induced by NHS mutations. **(A)** Volcano plot of Site mutation (c.3847C>T) vs wild-type. Red: 229 upregulated genes (log_2_FC>1, FDR<0.05); Green: 841 downregulated genes (log_2_FC<-1, FDR<0.05); Gray: non-significant genes; **(B)** Volcano plot of Del mutation (c.2519_2520del) vs wild-type. Red: 81 upregulated genes; Green: 699 downregulated genes (same thresholds as A). Dashed lines indicate significance cutoffs.

**Table S1.** Primers for sgRNA vector construction

| **Primer** | **Sequence 5’-3’** |
| --- | --- |
| Site mutations sgRNA-F | GCTATTTCTAGCTCTAAAACAGTGCGTACAGAGACTGAG |
| Site mutations sgRNA-R | TGTGGAAAGGACGAAACACCGCTCAGTCTCTGTACGCACT |
| Site-ssODN-donor | CCGCCCAAATGATTTGGATGGTAAAATAATACAATATGGACCTGGTCCAGACGAAACTCTAGAACAGGTATAGAAGGCACCCTCTGCAGGTCTGGAGGAAGTTGCACAACCTGAATCTGTGGATGTAATCACATCTCAGTC |
| Del mutations sgRNA-F | GCTATTTCTAGCTCTAAAACAGCATCTCTTTCAGGAAACC |
| Del mutations sgRNA-R | TGTGGAAAGGACGAAACACCGGTTTCCTGAAAGAGATGCT |
| Del-ssODN-donor | CCAAGCATCTCTTTCAGGAAACCAAAGGCAAAGCCGACCCCACCTAAACGTAGCTCATCATTGAGGAAGTGATGGAAACGCAGATATTTCTGAGAAGAAAGAACCAAAGATAAGCAGTGGTCAGCACCTGCCTCACAGTT |

**Table S2.** Primers used for Sanger sequencing analysis.

| **Primer** | **Sequence 5’-3’** |
| --- | --- |
| Site mutations YZ-F | GATGGGACCAGATAAACTACA |
| Site mutations YZ-R | TTGGGTCTCCTCTGCTTTG |
| Del mutations YZ-F | AGACAAAGCGGACACTAGCT |
| Del mutations YZ-R | TCAAGTCATTTAGGAGCCAGA |

**Table S3.** Primers used for quantitative real-time PCR.

| **Primer** | **Sequence 5’-3’** |
| --- | --- |
| NHS-qF | GAGCTCGAGAGCGACATCC |
| NHS-qR | GGTTGGAGACGGGCACTG |
| COL4A2-qF | GCGGCTTACACGGCTTGC |
| COL4A2-qR | CTCTCCTGGGTCACCTCTTTCC |
| MMP2-qF | GGACGGACTCCTGGCTCATG |
| MMP2-qR | CCATACTTCACACGGACCACTTG |
| BAX-qF | TGCGTCCACCAAGAAGCTGAG |
| BAX-qR | TCCACGGCGGCAATCATCC |
| BCL2-qF | CGAGTGGGATGCGGGAGATG |
| BCL2-qR | CGGGATGCGGCTGGATGG |
| ALDH3A1-qF | TCCAGCAACGACAAGGTGATTAAG |
| ALDH3A1-qR | GCAAGGTGATGTGGACGATGAC |
| SOD2-qF | GCCCTGGAACCTCACATCAAC |
| SOD2-qR | CGCCTCCTGGTACTTCTCCTC |
| PGC-1α-qF | TTCCAGGTCAAGATCAAGGTCTCC |
| PGC-1α-qR | TGCGTGCGGTGTCTGTAGTG |
| CDKN1A-qF | ACCCTTGTGCCTCGCTCAG |
| CDKN1A-qR | CGGCGTTTGGAGTGGTAGAAATC |
